# TGF-β Signaling Regulates Cementum Formation through Osterix Expression

**DOI:** 10.1038/srep26046

**Published:** 2016-05-16

**Authors:** Hwajung Choi, Yu-Hyun Ahn, Tak-Heun Kim, Cheol-Hyeon Bae, Jeong-Chae Lee, Hyung-Keun You, Eui-Sic Cho

**Affiliations:** 1 Cluster for Craniofacial Development and Regeneration Research, Institute of Oral Biosciences, Chonbuk National University School of Dentistry, Jeonju 54896, South Korea; 2 Department of Periodontology, School of Dentistry, Wonkwang University, Iksan 54538, South Korea

## Abstract

TGF-β/BMPs have widely recognized roles in mammalian development, including in bone and tooth formation. To define the functional relevance of the autonomous requirement for TGF-β signaling in mouse tooth development, we analyzed *osteocalcin-Cre* mediated *Tgfbr2* (*OC*^*Cre*^*Tgfbr2*^*fl/fl*^) conditional knockout mice, which lacks functional TGF-β receptor II (TβRII) in differentiating cementoblasts and cementocytes. Strikingly, *OC*^*Cre*^*Tgfbr2*^*fl/fl*^ mutant mice exhibited a sharp reduction in cellular cementum mass with reduced matrix secretion and mineral apposition rates. To explore the molecular mechanisms underlying the roles of TGF-β signaling through TβRII in cementogenesis, we established a mouse cementoblast model with decreased TβRII expression using OCCM-30 cells. Interestingly, the expression of osterix (Osx), one of the major regulators of cellular cementum formation, was largely decreased in OCCM-30 cells lacking TβRII. Consequently, in those cells, functional ALP activity and the expression of genes associated with cementogenesis were reduced and the cells were partially rescued by Osx transduction. We also found that TGF-β signaling directly regulates Osx expression through a Smad-dependent pathway. These findings strongly suggest that TGF-β signaling plays a major role as one of the upstream regulators of Osx in cementoblast differentiation and cementum formation.

Cementum, a key component of the periodontium, is a mineralized tissue layer covering the tooth root that plays an essential role in anchoring teeth to the surrounding alveolar bone^1^. The integrity of cementum is crucial for tooth stability and for protecting tooth from resorption. Periodontal regeneration aims to recapitulate the crucial stages of development in order to restore lost tissues to their original form and function^2^.

Cementoblasts, the cells forming cementum at the lining along the tooth root surface, are functionally responsible for cementum matrix deposition and mineralization^3^. Cementoblasts share many properties with osteoblasts, including the expression of genes such as runt-related gene 2 (Runx2), osterix (Osx), type I collagen (Col1), osteocalcin (Ocn), osteopontin (Opn) and bone sialoprotein (Bsp) and the ability to promote mineral nodules *in vitro*^4^,^5^. However, the molecular factors and local conditions directing a progenitor cell toward the osteoblastic or cementoblastic lineage may be different. Thus, a full understanding of the tissue is paramount for development of therapies and periodontium regeneration. However, the exact molecular mechanisms regulating cementoblast differentiation and cementum formation have not been fully elucidated.

The transcription factor Osx is required for osteoblast differentiation and bone formation during embryonic development and postnatal bone growth and homeostasis^6^,^7^, as well as for regulation of tooth root formation^8^. Recent studies have demonstrated as crucial role of Osx in cementoblast differentiation and cementum formation during tooth development^9^,^10^. The studies have shown a close relationship between the spatiotemporal expression pattern of Osx and formation of cellular cementum. Furthermore, Osx overexpression *in vivo* is associated with accelerated cementum formation^9^. In addition, conditional deletion of *Osx* in mesenchymal cells led to a sharp reduction in cellular cementum formation, including cementum mass and mineral deposition rate^9^. This finding sheds light on the mechanisms by which Osx regulates cementoblast differentiation *in vivo* and *in vitro*. With respect to explore the mechanisms of Osx control of cementogenesis, it has been demonstrated that Osx enhances cementoblast differentiation but inhibits cell proliferation by reducing Wnt pathway signaling via positive regulation of DKK1 in cementoblasts^10^. However, the upstream regulators of Osx in cementoblast differentiation and cementum formation remain unclear.

TGF-βs have widely recognized roles in hard tissue formation during mammalian development and exhibit versatile regulatory functions in the body^11^,^12^. Recent advances in molecular and genetic studies using gene targeting in mice have enable a better understanding of TGF-β signaling in bone and in the signaling networks underlying osteoblast differentiation and bone formation^13^,^14^,^15^,^16^. TGF-β regulates the proliferation and differentiation of osteoblasts both *in vitro* and *in vivo*; however, the effects of TGF-β on osteoblast differentiation depend on the extracellular milieu and the differentiation stage of the cells^17^. TGF-β stimulates proliferation and early osteoblast differentiation while inhibiting terminal differentiation^17^,^18^. However, the net effects of postnatal TGF-β signaling on cementoblast differentiation and cementum formation remain unclear.

In this study, we demonstrated the role of TGF-β signaling in cementogenesis using a conditional knockout mouse and *in vitro* cellular approaches. Our observations suggest that TGF-β signaling plays a major role in establishing the correct formation of cellular cementum through regulation of Osx expression.

## Results

### Targeted inactivation of TβRII in cementoblasts results in a sharp reduction in cellular cementum

**Figure 1:**
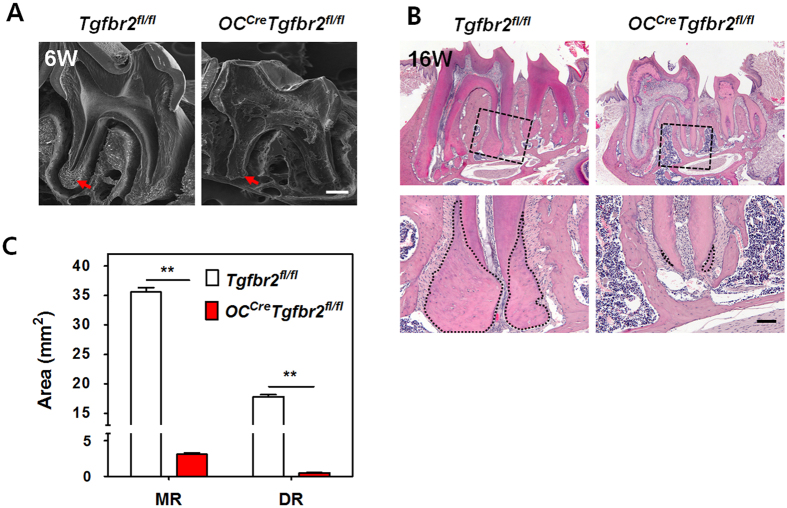
A sharp reduction in cellular cementum of mandibular molars of *OC*^*Cre*^*Tgfbr2*^*fl/fl*^ mice. (**A**) SEM analysis of second molars from *Tgfbr2*^*fl/fl*^ and *OC*^*Cre*^*Tgfbr2*^*fl/fl*^ mice at 6 weeks of age (6W). Red arrows show the sharp reduction in apical cellular cementum in differentiating cementoblasts of TβRII-inactivated mice *in vivo*. Scale bar, 200 μm. (**B**) The upper images show representative histological H-E-stained sections of the mandibular molars from *Tgfbr2*^*fl/fl*^ and *OC*^*Cre*^*Tgfbr2*^*fl/fl*^ mice at 16 weeks of age (16W). Below are higher-magnification views of the boxed area from the upper images. Scale bar, 100 μm. (**C**) Quantification of the cellular cementum area of the mandibular first molars indicated in panel B. The data represent the mean ± SE of three measurements of five separate representative slides from each group. **P < 0.01. MR, mesial root; DR, distal root.

To study the role of TGF-β signaling in *in vivo* differentiation of cementoblasts, we generated mice conditionally inactive for *Tgfbr2*, the gene encoding TβRII. *Tgfbr2*^*fl/fl*^ mice, having *loxP* sites at introns 1 and 2 of *Tgfbr2*^19^, were crossed with mice expressing Cre recombinase under the control of the mouse *osteocalcin* (*OC*) promoter^20^. We previously reported LacZ expression in the teeth of *ROSA26 OC-Cre* double-transgenic mice. We confirmed that the Cre recombinase produced from the *OC-Cre* promoter was active in differentiating cementoblasts and cementocytes of the developing tooth^21^. Interestingly, conditional *Tgfbr2* knockout (*OC*^*Cre*^*Tgfbr2*^*fl/fl*^) mice displayed a sharp reduction in cellular cementum mass compared to the WT controls (*Tgfbr2*^*fl/fl*^), as observed by SEM at 6 weeks of age (Fig. 1A). Furthermore, H-E staining of tissue sections of the mandibular first molars at 16 weeks of age confirmed the tooth phenotypes following further development (Fig. 1B). Dramatic changes in the gross cellular cementum area of the mandibular first molar were observed, as shown in the measured areas of the mesial root (MR) and distal root (DR) (Fig. 1C). There was a largely reduced cementum area (approximately 2% to 8% of WT control) in both roots of the *OC*^*Cre*^*Tgfbr2*^*fl/fl*^ mice, while no remarkable difference in total root length was observed between WT control and conditional knockout mice.

### Reduced matrix formation and mineralization rates of cellular cementum with inactivation of TβRII in cementoblasts

**Figure 2:**
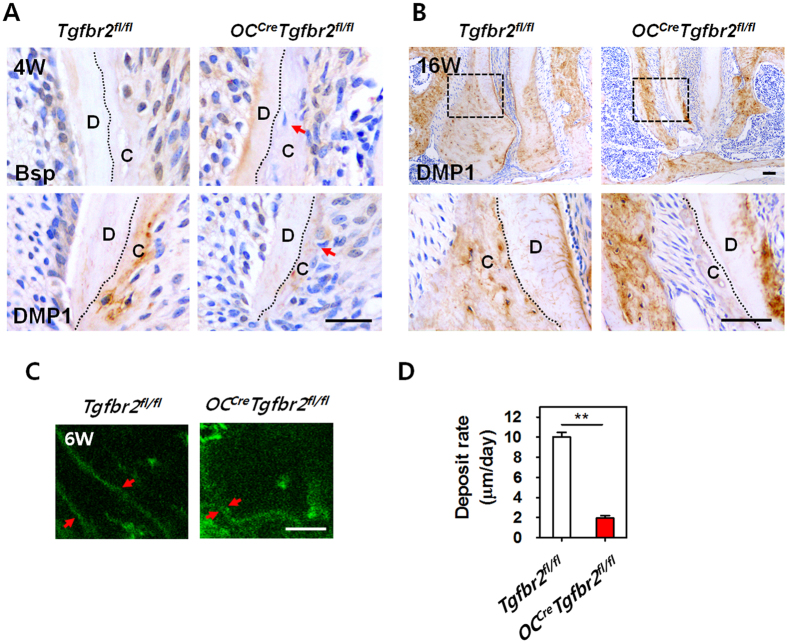
Reduced matrix formation and mineralization rates in cellular cementum of mandibular molars of *OC*^*Cre*^*Tgfbr2*^*fl/fl*^ mice. (**A**) Immunohistochemical staining analysis of Bsp and DMP1 in mandibular first molars from *Tgfbr2*^*fl/fl*^ and *OC*^*Cre*^*Tgfbr2*^*fl/fl*^ mice at 4 weeks of age (4W). Red arrows indicate reduced expression of Bsp and DMP1 in cementocytes and in the developing cementum mass in *OC*^*Cre*^*Tgfbr2*^*fl/fl*^ mice compared with *Tgfbr2*^*fl/fl*^ mice. Scale bar, 25 μm. (**B**) At 16 weeks of age (16W), the differences in the volumes and DMP1-staining intensities of the cementum masses between *Tgfbr2*^*fl/fl*^ and *OC*^*Cre*^*Tgfbr2*^*fl/fl*^ mice are more remarkable as a result of matrix accumulation in WT cementum (Up). Below are higher-magnification views of the boxed area in the upper images. Scale bar, 50 μm. (**C**) Red arrows show the distance of fluorochrome labeling lines indicating that the mineralization rate was decreased in *OC*^*Cre*^*Tgfbr2*^*fl/fl*^ mice compared with *Tgfbr2*^*fl/fl*^ mice at 6 weeks of age (6W). Scale bar, 25 μm. (**D**) Quantification of the mineral deposition rates in cementum of the mandibular first molars indicated in panel (**C**). The data represent the mean ± SE of three measurements of two separate representative slides from each group. **P < 0.01. (**C**), cementum; (**D**), dentin.

We next asked whether the inactivation of TβRII in cementoblasts altered the levels of extracellular matrix proteins and mineralization important for the regulation of cellular cementum. As described previously, cellular cementum is formed by Osx-positive mesenchymal progenitor cells or cementoblasts in the apical root^9^. Osx was found to be expressed in cementoblasts and cementocytes at 3 weeks postnatally, and then its expression increased sharply 4 to 6 weeks postnatally; these observations correlate closely with the formation of cellular cementum^8^. Immunohistochemical staining showed that Bsp and DMP1, key markers of cementum, were highly expressed in the cementocytes of the developing cementum in *Tgfbr2*^*fl/fl*^ mice at 4 weeks. Inactivation of TβRII in cementoblasts led to dramatic reductions in Bsp and DMP1 in the cementocytes and in cementum mass in *OC*^*Cre*^*Tgfbr2*^*fl/fl*^ mice (Fig. 2A). Moreover, it was remarkable that the expression level of DMP1 was reduced in the cementocytes with a sharp reduction in cementum mass in *OC*^*Cre*^*Tgfbr2*^*fl/fl*^ mice at 16 weeks of age (Fig. 2B). To address whether TβRII plays a role in controlling the mineralization rate in cementogenesis, a fluorochrome labeling assay was used. As shown in Fig. 2C, the fluorochrome labeling lines appeared thicker in the cementum area of *Tgfbr2*^*fl/fl*^ mice compared to *OC*^*Cre*^*Tgfbr2*^*fl/fl*^ mice. The distance between the two lines, reflecting the rate of cellular cementum formation, was much shorter in *OC*^*Cre*^*Tgfbr2*^*fl/fl*^ mice (1.9 μm/day) than in *Tgfbr2*^*fl/fl*^ mice (10.0 μm/day) (Fig. 2D).

### Inactivation of TβRII results in reduced Osx expression and impaired differentiation of cementoblasts

**Figure 3:**
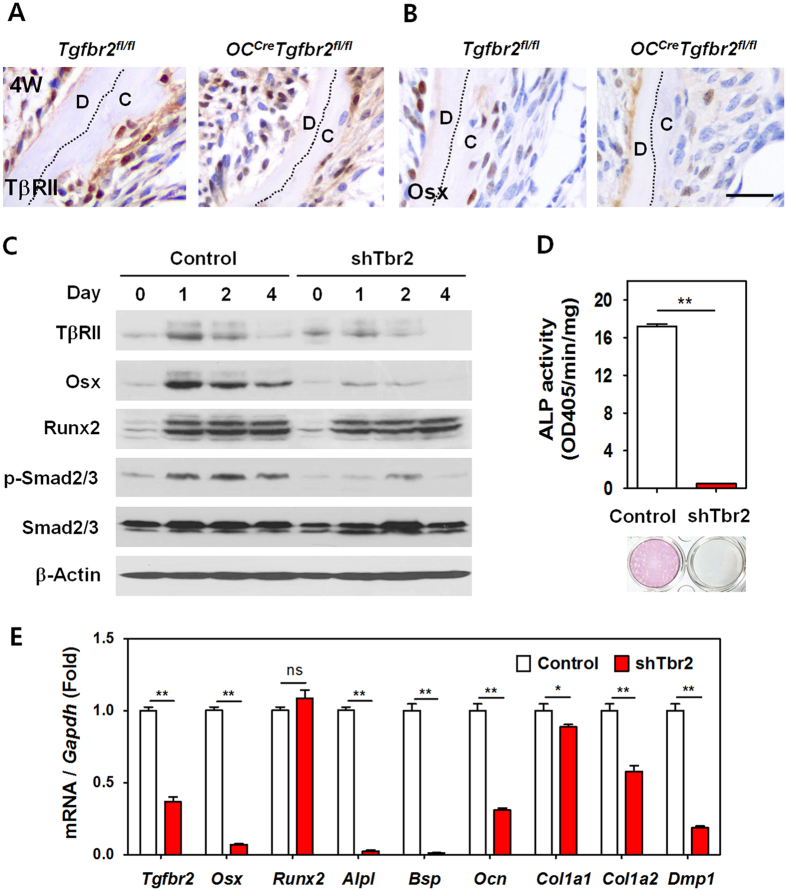
Inactivation of TβRII results in reduced Osx expression and impaired differentiation of cementoblasts. Immunohistochemical staining analysis of TβRII (**A**) and Osx (**B**) expression in mandibular first molars from *Tgfbr2*^*fl/fl*^ and *OC*^*Cre*^*Tgfbr2*^*fl/fl*^ mice at 4 weeks of age (4W). Scale bar, 25 μm. (**C**) Western blot analysis of the indicated molecules in OCCM-30 cells stably expressing either control or mouse *Tgfbr2* shRNA (shTbr2) following the indicated duration of OM treatment. Cropped images were displayed here and the original full-length blots were presented in Supplementary Figure 1. The samples were derived from the same experiment and gels/blots were processed under the same experimental conditions. β-Actin was used as a loading control. (**D**) Enzymatic activity of ALP in control and shTbr2 OCCM-30 cells (Upper graph). The data represent the mean ± SE of three measurements in each group. **P < 0.01. Lower image shows that OCCM-30 cells lacking functional TβRII have very low degree of ALP staining when compared with control cells. (**E**) Real-time qPCR analysis for the assessment of functional cementoblast differentiation in control and shTbr2 OCCM-30 cells after OM treatment for 4 days. The data represent the mean ± SE of three measurements in each group. **P < 0.01, *P < 0.05. (**C**), cementum; (**D**), dentin.

There was a definite decrease in TβRII expression in the cementum-lining cells of *OC*^*Cre*^*Tgfbr2*^*fl/fl*^ mice at 4 weeks of age (Fig. 3A). However, not all of the cementum-lining cells were TβRII-negative; some had intact TβRII, implying that OC-negative cells exist along with OC-positive cementoblast progenitor cells in the lining. We noticed that the sharp reduction in cementum mass in *OC*^*Cre*^*Tgfbr2*^*fl/fl*^ mice was very similar to that obtained with a conditional deletion of *Osx* using the 2.3-kb *Col1a1*-Cre^9^. We hypothesized that deletion of TβRII inhibits cementoblast differentiation partly through a decrease in Osx protein. As shown in Fig. 3B, the number of Osx-positive cells was obviously decreased in the cellular cementum of *OC*^*Cre*^*Tgfbr2*^*fl/fl*^ mice at 4 weeks of age. To investigate the molecular mechanism of altered TGF-β signaling in cementogenesis, we employed OCCM-30 cementoblast-like cells, which were derived from OC-positive root-surface cells of a developing mouse tooth and have been reported to display cementoblast-like characteristics^5^. To determine whether the clonally derived OCCM-30 cell line is a suitable model of cementogenesis, we investigated its temporal sequence of development in culture and examined the time course of extracellular matrix accumulation (Supplementary Figure 2). This cementoblast-like cell system progressed through distinct stages of cementoblast development in a manner analogous to *in vivo* cementum formation, as demonstrated by the temporal sequence of development characterized by distinct initial proliferation, intermediate differentiation, and terminal matrix-formation stages. The initial stage of development was characterized by cell proliferation as evidenced by high level of Cyclin D1 that decreased sharply after OM treatment. Importantly, the intermediate phase was defined as a differentiation stage evidenced by sharp increases in transcriptional factors, including Osx and Runx2, which participate in the processes of both osteogenesis and odontogenesis^6^,^8^,^22^. The final phase of OCCM-30 maturation was defined by matrix formation with progressive increases in extracellular matrix proteins including Bsp and Opn. Based on this observation, we established OCCM-30 cementoblasts lacking functional TβRII (shTbr2) by stably expressing shRNA for mouse *Tgfbr2* (Supplementary Figure 3A). Inactivation of TβRII in OCCM-30 cells significantly decreased the cell proliferation rate (Supplementary Figure 3B). Morphologically, the original OCCM-30 cells remained round while the shTbr2 cementoblasts developed a larger, elongated and spindle-like shape. Furthermore, these morphological changes showed a much clearer distinction upon differentiation by OM treatment for 4 days (Supplementary Figure 3C).

Western blot analysis revealed consistently lower expression of Osx in shTbr2 OCCM-30 cells compared with the expression seen in the controls at various time points of differentiation while no difference in the expression of Runx2 was observed (Fig. 3C). Studies using mice lacking the *Alpl* gene have demonstrated that ALP is required for proper cementum formation^23^. We found that the enzymatic activity of ALP, as measured by ALP staining, was nearly non-existent in shTbr2 cementoblasts (Fig. 3D). The level of *Alpl* gene expression was also decreased in shTbr2 cementoblasts, as determined by real-time qPCR (Fig. 3E). To examine the status of cementoblast functional differentiation, we assayed the expression of other genes using real-time qPCR. The mRNA levels of *Osx* and other genes associated with cementogenesis were significantly lower in shTbr2 cementoblasts than in the controls, with the exception of no observed significant difference in Runx2 expression (Fig. 3E). Moreover, the expression levels of the genes for extracellular matrix proteins, including Bsp (*Bsp*), Ocn (*Ocn*), collagen type I (*Col1a1* and *Col1a2*), and DMP1 (*Dmp1*), were all reduced as a result of decreased TβRII expression in cementoblasts.

### Enforced Osx expression partially rescues the impaired differentiation of cementoblasts resulting from inactivation of TβRII

**Figure 4:**
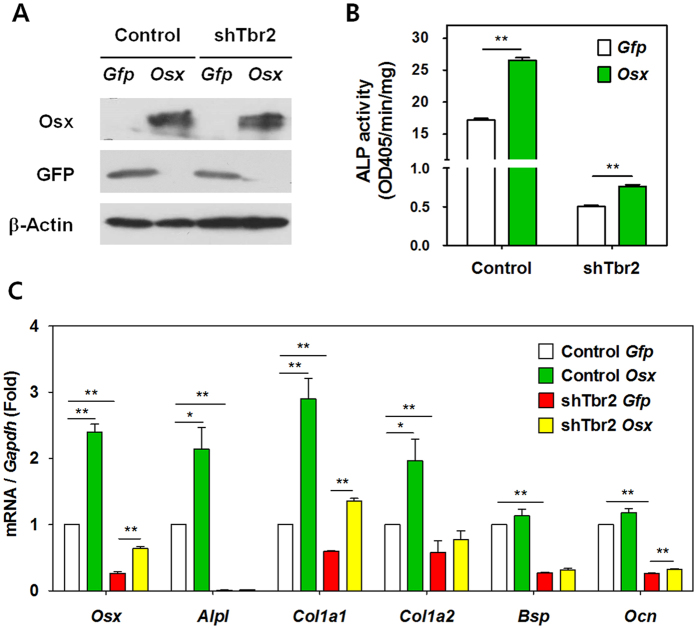
Enforced Osx expression partially rescues impaired cementoblast differentiation and reduced ALP activity following inactivation of TβRII. (**A**) Western blot analysis to confirm enforced Osx expression in control and shTbr2 OCCM-30 cells after transfection of *Osx* or *Gfp* construct. Cropped images were displayed here and the original full-length blots were presented in Supplementary Figure 4. The samples were derived from the same experiment and gels/blots were processed under the same experimental conditions. β-Actin was used as a loading control. (**B**) Partial rescue of the impaired ALP activity in shTbr2 OCCM-30 cell by enforced Osx expression. The data represent the mean ± SE of three measurements in each group. **P < 0.01. (**C**) Real-time qPCR analysis of the indicated genes in control and shTbr2 OCCM-30 cells after OM treatment for 4 days after transfection. The data represent the mean ± SE of three measurements in each group. **P < 0.01, *P < 0.05.

*OC*^*Cre*^*Tgfbr2*^*fl/fl*^ mice display a striking similarity to mice with a conditional deletion of *Osx* established with the 2.3-kb *Col1a1*-Cre with regard to the absence of a statistically significant reduction in *Osx* levels, suggesting that TGF-β signaling regulates Osx expression via a transcriptional mechanism. To test this hypothesis, Osx expression was enforced in control and shTbr2 OCCM-30 cells by retroviral infection. Equivalent expression of Osx was achieved in both cell types (Fig. 4A). Although ALP activity was not fully recovered by enforced Osx expression in shTbr2 OCCM-30 cells, Osx expression promoted significant ALP activity in both OCCM-30 control and shTbr2 cells (Fig. 4B). Similarly, enforced Osx expression significantly induced the expression of cementogenesis-associated genes, including *Alpl*, *Col1a1*, *Col1a2*, in control cementoblasts. The mRNA expression levels of Osx target genes were partially recovered by enforced Osx expression in shTbr2 OCCM-30 cells. However, *Alpl* and *Bsp* expression was not recovered, implying that they have other upstream regulators (Fig. 4C). Therefore, when highly expressed via viral transduction at equivalent levels in control and shTbr2 OCCM-30 cells, Osx expression could partially rescue the impaired differentiation of cementoblasts resulting from inactivation of TβRII.

### TGF-β signaling regulates Osx expression through a Smad-dependent pathway in cementoblasts

**Figure 5:**
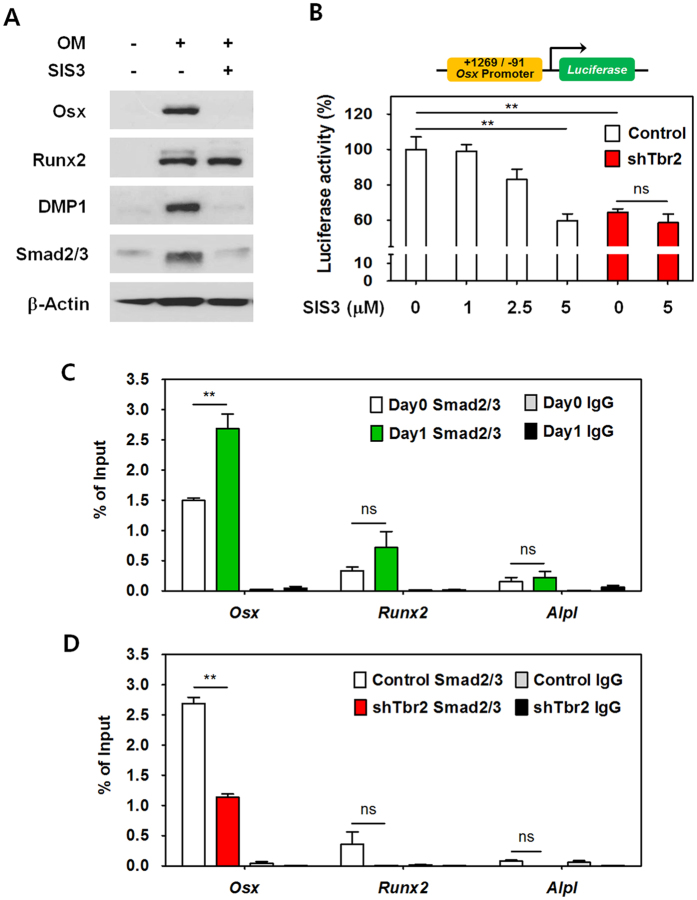
TGF-β signaling regulates Osx expression during cementoblast differentiation through a Smad-dependent pathway. (**A**) Western blot analysis of the indicated molecules in OCCM-30 cells treated with SIS3, an inhibitor of Smad3, during OM treatment for 4 days. Cropped images were displayed here and the original full-length blots were presented in Supplementary Figure 5. The samples were derived from the same experiment and gels/blots were processed under the same experimental conditions. β-Actin was used as a loading control. (**B**) Luciferase reporter activities of the *Osx* promoter ( + 1269 to −91) in control and shTbr2 OCCM-30 cells treated with SIS3 at the indicated concentration. The data represent the mean ± SE of three measurements in each group. **P < 0.01. ns, not significant. (**C**) ChIP-qPCR analysis performed using an anti-Smad2/3 antibody and chromatin from OCCM-30 cells treated with OM for 0 or 1 day, respectively. (**D**) ChIP-qPCR analysis using an anti-Smad2/3 antibody and chromatin from control and shTbr2 OCCM-30 cells after 1 day of OM treatment. The data represent the mean ± SE calculated of three measurements in each group. **P < 0.01, ns, not significant.

The TGF-β system transmits signals via Smad mediators to regulate a variety of biological processes. The rescue of TβRII-deficient cementoblasts by enforced Osx expression suggests that Osx expression is regulated through Smad2/3 downstream of TGF-β signaling and is responsible for at least some of its effects in promoting cementoblast differentiation. To determine whether the regulation of Osx expression by TβRII is dependent on Smad mediators, we examined the involvement of Smad3 in Osx expression using a pharmacological inhibitor of Smad3, SIS3^24^. As shown in Fig. 5A, expression of Osx and DMP1 induced by OM treatment decreased sharply upon Smad3 inhibition by SIS3, while no difference in Runx2 was observed. To determine whether Smad3 regulates *Osx* transcription, *Osx* promoter activity was also examined using SIS3 in control and shTbr2 OCCM-30 cells. As shown in Fig. 5B, *Osx* promoter activity in control cementoblasts was decreased in a concentration-dependent manner by SIS3. Most significantly, treatment of control OCCM-30 cells with 5 μM SIS3 resulted in *Osx* promoter activity that was as low as the basal level of promoter activity in shTbr2 OCCM-30 cells. As expected, no significant difference was seen in shTbr2 OCCM-30 cells treated with SIS3.

It is still unclear which mechanism mediates transcriptional regulation of *Osx* during cementoblast differentiation. To address the hypothesis that the Smad2/3 downstream effectors of TGF-β signaling regulate transcription through direct binding to the promoter region of *Osx*, we performed ChIP-qPCR analysis using OCCM-30 cells treated with OM. As shown in Fig. 5C, significantly increased recruitment of Smad2/3 to the regulatory regions of *Osx* was observed in OCCM-30 cells treated for 1 day with OM, compared with untreated cells. However, the association of Smad2/3 with the regulatory regions of *Runx2* was modest and not statistically significant. Additionally, no association with the regulatory regions of *Alpl* was detected upon OM treatment for 1 day. As expected, the association of Smad2/3 with the regulatory regions of *Osx* was significantly decreased in shTbr2 cementoblasts compared with control cells, and no significant associations with the regulatory regions of *Runx2* and *Alpl* were observed (Fig. 5D). This ChIP-qPCR data supports the concept that TβRII regulates *Osx* expression directly through the interaction with Smad2/3 in the regulatory regions of *Osx*, while no direct binding in the regulatory regions of *Runx2* and *Alpl* was observed.

## Discussion

Molecular and genetic animal models established with spatiotemporal gene targeting technologies are useful in studies evaluating critical developmental factors, including growth factors, signaling molecules and transcriptional factors, in order to overcome disadvantages such as death *in utero*. In the present study, we analyzed mice that having *loxP* sites at introns 1 and 2 of *Tgfbr2* under the control of mouse *osteocalcin* promoter. We found a sharp reduction in cellular cementum in developing teeth. Interestingly, these phenotypes are very similar to those observed with a conditional deletion of *Osx* with the 2.3-kb *Col1a1*-Cre in the absence of a statistically significant reduction in *Osx* levels, as previously reported^9^. In addition to an *in vivo* model, *in vitro* cementoblast models showed that endogenous TGF-β signaling controls cementoblast differentiation by directly regulating Osx expression and that this process is mediated through a Smad signaling pathway. The expression of Osx in cementoblasts lacking TβRII was largely reduced; conversely, enforced expression of Osx partially rescued cells lacking TβRII *in vitro*. The current study using immunohistochemistry assays to evaluate the protein expression levels of DMP1 and Bsp in tissues from TβRII conditional knockout mice, coupled with *in vitro* assays using cementoblasts with a shRNA system, supports a similar mechanism by which TGFβ signaling controls cementogenesis via the transcriptional activity of Osx.

Osx was originally defined as a zinc finger-containing transcription factor essential for osteoblast differentiation and bone formation during bone homeostasis^6^,^7^. Recent studies have demonstrated that Osx accelerates cementoblast differentiation in part through down-regulation of Wnt/β-catenin signaling by activating DKK1^10^. Recently, we also reported that Osx regulates odontoblast differentiation, maturation and root elongation^8^,^25^. These findings strongly indicate that Osx is a site-specific regulator in tooth root formation. The regulation of Osx in osteogenesis has been documented using various *in vitro* and *in vivo* models. Osx expression is activated by BMPs through Runx2-dependent and -independent mechanisms involving Dlx5 and Msx2^26^,^27^. These mechanistic reports uncovered both genetic and epigenetic mechanisms of regulation of Osx expression. These studies also showed that BMP-2 induction of Osx required the transcriptional activation of Dlx5 by p38 MAPK-mediated phosphorylation^27^,^28^. Moreover, additional pathways may act in parallel to, or independent of Osx transcription to post-transcriptionally modulate the function of Osx through phosphorylation, regulation of protein stability, and transcriptional activity^29^,^30^,^31^. However, in contrast to our understanding of bone biology with BMPs, the molecular mechanisms by which the expression of Osx can be regulated by TGF-β in cementogenesis remain unclear. In the present study, we first documented that Osx expression during cementoblast differentiation and cementum formation might be controlled directly by endogenous TGF-β signaling.

TGF-β is a potent multifunctional regulator of cell growth and differentiation. Although nearly all cells synthesize and respond to TGF-β, hard tissues including bone and cartilage are particularly rich in this growth factor. In previous studies, mammalian TGF-β isoforms have osteoinductive activity^32^,^33^,^34^,^35^, whereas transgenic mice overexpressing TGF-β2 in bone exhibited an osteoporotic phenotype characterized by increased osteoblast and osteoclast activities and impaired matrix mineralization by osteoblasts^36^. It is well established that TGF-β signaling suppresses osteoblast differentiation and maturation *in vivo* and *in vitro*^13^,^14^,^15^. As reported previously with a mouse model for endogenous TGF-β signaling in osteoblasts, dominant negative inhibition of TGF-β responsiveness under the control of the rat osteocalcin promoter fragment leads to decreased bone remodeling and increased trabecular bone mass^13^. Exogenous TGF-β also exerts inhibitory effects on cementoblast differentiation, as evidence by decreased Bsp and Ocn expression *in vitro* as previously described^37^. However, the roles of endogenous TGF-β in cementum formation have not been fully elucidated. Despite the similar properties of osteoblasts and cementoblast *in vitro*, the discrepancy between the physiological roles of TGF-β in bone development and cementum formation in tooth development may be due to differences in the complexity of the *in vivo* physiological environment and developmental status. The effects of TGF-β on osteoblast differentiation depend on the extracellular milieu and the differentiation stage of the cells^17^. Furthermore, the anabolic signaling network for the coupling of bone resorption and formation is orchestrated by the interaction of local osteotropic factors, such as TGF-β and insulin-like growth factor, with various systemic hormones like PTH^16^. Although osteoblasts and cementoblasts have many features in common and similar regulation mechanisms, cementum seems to have unique properties during postnatal development and regulation. There is little evidence for cementum remodeling, while bones are continuously formed and resorbed through a process of bone remodeling. A system consisting of osteoblasts, osteocytes, bone-lining cells, and osteoclasts is required for proper functioning of bone^3^. However, cementum seems to function without such a complex cell system, with signals from the adjacent periodontal ligament likely influencing cementoblast function.

During tooth development, Runx2 and Osx are both highly expressed in the dental mesenchyme at early stages during which the crown develops. However, from the bell stage to the postnatal stage, only Osx was expressed during root developments, while the expression of Runx2 declined sharply^38^. When mice haploinsufficient for Runx2 were examined in order to characterize its function on cementum, the results were found to be normal, even in the presence of serious bone defects^39^. Moreover, patients with cleidocranial dysplasia, caused by mutations involving the *Runx2* gene in humans, show no statistically significant difference in cementum formation compared with normal subjects^40^. These findings support the idea that Runx2 functions primarily embryonically at earlier stages in tooth development, while Osx participates at later stages, specifically in tooth root development, including the cementum formation stage. Interestingly, the expression level of Osx was sensitively altered by the modulation of TGF-β signaling by treatment with shRNA for *Tgfbr2*, while the gross level of Runx2 was not altered. Given the central role of TGF-β signaling in Osx expression during cementogenesis, these results indicate that the regulation of Osx expression is mechanically more critical than that of Runx2 during cementogenesis.

In summary, we demonstrated that *OC*^*Cre*^*Tgfbr2*^*fl/fl*^ mice, with conditionally disrupted TβRII in cementoblasts during tooth development, exhibit a sharp reduction in cellular cementum. Furthermore, the functional lack of TβRII in cementoblasts accompanies a decrease in the expression of Osx, a key regulator of cellular cementum formation, and consequently results in decreased ALP activity and decreased expression of genes associated with cementogenesis *in vitro*. These results demonstrate a direct effect of TGF-β signaling on endogenous cementum formation. Furthermore, the mechanistic analysis indicates that TGF-β signaling controls cementoblast differentiation by directly regulating Osx expression through a Smad-dependent pathway. Our findings provide important information for understanding the mechanism of cementoblastic differentiation during tooth root formation, thereby supporting the future development of therapies and periodontium regeneration strategies.

## Materials and Methods

### Mouse strain and tissue preparation

All procedures were performed in accordance with the National Institutes of Health Guidelines on the Use of Laboratory Animal. Experimental protocols and animal care methods were approved by the Animal Welfare Committee of Chonbuk National University. *OC*^*Cre*^*Tgfbr2*^*fl/fl*^ mice were generated and genotyped as described previously^41^. For histologic analysis, mice were sacrificed, and their mandibles were carefully dissected. The tissues were fixed in 4% paraformaldehyde and decalcified in 10% ethylenediaminetetraacetic acid solution for 2 to 4 wk at 4 °C. The decalcified tissues were dehydrated through a graded ethanol series, embedded in paraffin, and sectioned at 5-μm thickness. Slides were stained with hematoxylin and eosin (H-E) for observation and further analysis. For statistical analysis, six independent littermates were used in each study.

### Immunohistochemistry

Immunohistochemistry was performed as described previously^42^. Briefly, sections were treated with 3% hydrogen peroxide, and incubated with rabbit polyclonal antibodies against Osx (1:200; Santa Cruz Biotechnology, Inc., Dallas, TX, USA), pSmad2/3 (1:500; Maine Medical Center Research Institute, Scarborough, ME, USA), TβRII (1:200; Abcam, Cambridge, MA, USA), BSP (1:200; Abcam) and DMP1 (1:750; TaKaRa Bio, Shiga, Japan). The Histostain Plus rabbit primary (DAB) kit (Zymed Laboratories, San Francisco, CA, USA) was used according to the manufacturer’s instructions. The average cellular cementum area was calculated using three measurements of five representative individual slides in each group at the age of 16 weeks using the analySIS Pro imaging system (Soft Imaging System, Münster, Germany).

### Double-fluorochrome labeling

Six-week-old animals were injected with calcein (20 mg/kg of body weight intraperitoneally; Sigma-Aldrich) at 7 and 2 days before being sacrificed. Fifty-micrometer cross-sections were cut perpendicularly, passing through the midsection of the mandibular first molar, and were viewed under a model LSM510 confocal laser scanning microscope (Carl Zeiss, Ostalbkreis, Germany). We calculated the average cementum mineral apposition rate using eight separate measurements of the distance between the two fluorescent labels in each section using the analySIS Pro imaging system (Soft Imaging System) and then divided that measurement by the number of days between injections (5 days).

### Cell cultures

OCCM-30, a mouse cementoblast cell line, was kindly provided by Dr. Martha J. Somerman (National Institutes of Health, Bethesda, MD, USA) and cultured as described previously (2000-Employing a transgenic animal model to obtain cementoblasts *in vitro*). To induce cell differentiation and mineralization, 90–95% confluent cells were cultured in osteogenic medium (OM) containing growth media (GM) supplemented with 50 μg/ml ascorbic acid (Sigma Aldrich, St. Louis, MO, USA) and 10 mM β-glycerophosphates (Sigma Aldrich) for up to 7 days.

### Transfection and retroviral transduction

The full length open reading frame of mouse *Osx* (Accession No. NM_130458), cloned into the pCMV6 vector, was purchased from OriGene Technologies. A GFP construct was also transfected as a control. The luciferase *Osx* promoter plasmid + 1269/-91 was kindly provided by Dr. Mark Nanes (Emory University, Atlanta, GA, USA). Transfection experiments were performed with Lipofectamine^TM^ 3000 (Invitrogen) according to the manufacturer’s instructions. After 24 h, transfected cells were harvested for whole cell lysate preparation or cultured with OM for further differentiation. Viral particles were generated by transfecting a 293T-based amphotropic retroviral packaging cell line, Phoenix with a plasmid expressing shRNA for mouse *Tgfbr2* (OriGene Technologies, Rockville, MD, USA) using Lipofectamine^TM^ LTX and PLUS reagent (Invitrogen). Supernatants containing viral particles were collected between 48 and 72 h after transfection, filtered through a 0.45-μm filter, and used immediately. Subconfluent OCCM-30 cells were infected overnight with the retroviral particles expressing shRNA for mouse *Tgfbr2* in the presence of Polybrene. Forty-eight hours later, transduced OCCM-30 cells were selected from growth medium containing 10 μg/ml puromycin.

### ChIP-qPCR

ChIP assays were performed using a ChIP assay kit from Millipore (Billerica, MA, USA) according to the manufacturer’s protocol with minor modifications. In brief, cells were cross-linked by 1% formaldehyde for 5 min at room temperature. After a wash in phosphate buffered saline (PBS), an aliquot of the cross-linked chromatin was sonicated and incubated overnight with anti-Smad3 (1:100, Cell Signaling) or normal IgG. After incubation with protein G agarose beads for 2 h, and several washes, DNA–protein complexes were eluted. The cross-links were reversed by overnight incubation at 65 °C in the presence of 25 mM NaCl and subsequent digestion with RNase A and proteinase K, followed by purification of the ChIP-DNA. The ChIP-DNA was then amplified by qPCR; all reactions were carried out in triplicate. The sequences of the primers used for ChIP-qPCR are listed in Supplementary Table 1. The total amounts of binding by the Smad3 antibody were determined by expressing the amount of DNA obtained from each immunoprecipitation (IP) sample as a percentage of total DNA^43^.

### Alkaline phosphatase (ALP) activity and staining

Quantitative alkaline phosphatase activity was determined using an assay based on the hydrolysis of p-nitrophenylphosphate (p-NPP) to p-nitrophenol (p-NP). Cell layers were washed twice with ice-cold phosphate-buffered saline (PBS) and lysed in 50 mM Tris–HCl buffer (pH 7.0) containing 1% (v/v) Triton X-100 (Sigma Aldrich) and 1 mM PMSF (Sigma Aldrich). Whole cell lysates were assayed by adding 1 mg/ml of pNPP as a substrate in 0.1 M glycine buffer (pH 10.4) containing 1 mM ZnCl_2_ (Sigma Aldrich) and 1 mM MgCl_2_ (Sigma Aldrich) to each tube for 15 min at 37 °C. Reactions were stopped by adding NaOH (final 0.6 N), and the absorbance was measured spectrophotometrically at 405 nm. Enzyme activity was expressed as OD405/min/mg of protein or a percentage of control. The concentration of protein in each cell lysate was measured using a DC Protein Assay® (Bio-Rad Laboratories, Hercules, CA, USA). For ALP staining, cells were fixed in 10% formalin, incubated with 0.1% Triton X-100 for 5 min, and then stained with the Leukocyte Alkaline Phosphatase kit (Sigma Aldrich), according to the manufacturer’s protocol.

### Proliferation assay

Proliferation rates of OCCM-30 cells were measured using the Cell Counting Kit-8 (Dojindo Laboratories, Kumamoto, Japan) according to the manufacturer’s instructions. In brief, cells were cultured in 24-multiwell plates and treated with 10 μl/well of the kit solution. Absorbance was measured spectrophotometrically at 450 nm.

### RNA preparation and real-time qPCR

For preparation, 5.0 × 10^5^ cells were seeded in a 60 mm culture dish and cultured for 3 weeks under mineralization differentiation induction conditions. Total RNA was prepared using an RNeasy Mini kit (QIAGEN, Valencia, CA, USA) according to the manufacturer’s instructions, and cDNA was synthesized from 2 μg of total RNA using Superscript II reverse transcriptase (Invitrogen). Real-time PCR was performed with SYBR Green PCR Master Mix (Applied Biosystems, Warrington, Cheshire, UK) following the manufacturer’s protocols. Reaction conditions comprised 40 cycles of 15 sec of denaturation at 95 °C and 1 min of amplification at 60 °C. All reactions were run in triplicate, and expression was normalized to that of the housekeeping gene *glyceraldehyde-3-phosphate dehydrogenase* (*Gapdh*). Relative levels of transcript expression were quantified using the ΔΔ*Ct* method. The calculation was performed using the *Ct* value of *Gapdh* to normalize the *Ct* value of the target gene in each sample and obtain the Δ*Ct* value, which then was used to compare different samples. Relative mRNA expression was compared in a histogram. Specific primers sets used in the analysis are listed in Supplementary Table 2.

### Scanning electron microscopy (SEM)

The samples were fixed in 4% paraformaldehyde for 2 hours at room temperature and incubated in 8% formaldehyde for 2 days at 4 °C. The samples were dehydrated in an ascending ethanol series (30%, 50%, 70%, 80%, 90%, one time each and twice in 100%). After critical point drying, according to a standard procedure using liquid carbon dioxide, the samples were sputter-coated with platinum and examined with a scanning electron microscope (JSM-6400; JEOL, Tokyo, Japan) under 20-kV conditions.

### Luciferase activity

Luciferase activity was determined using the Dual-Luciferase reporter assay system (Promega), according to the manufacturer’s instructions. Light intensity was measured with a luminometer, and the luciferase activity was divided by that of the control reporter to normalize for transfection efficiency.

### Western blot analysis

Proteins (30 μg) were dissolved in sample buffer, and electrophoresis was carried out at a current of 25 mA for 2 h. Proteins were transferred from SDS-PAGE onto nitrocellulose membranes (Schleicher & Schuell, Dassel, Germany). Membranes were blocked for 1 h with 5% nonfat dry milk in PBS containing 0.1% Tween-20 (PBS-T) and incubated overnight with anti-Osx (Santa Cruz Biotechnology), TβRII (Santa Cruz Biotechnology), Runx2 (Abcam), DMP1 (TaKaRa Bio, Shiga, Japan), Bsp (Abcam), Opn (Abcam), Cyclin D1 (Santa Cruz Biotechnology) and β-Actin (Santa Cruz Biotechnology) IgG diluted in PBS-T buffer at 4 °C. After washing, the membranes were incubated with anti-rabbit or mouse-IgG conjugated horseradish peroxidase (Santa Cruz Biotechnology) for 1 h. Labeled protein bands were detected using an enhanced chemiluminescence system (Amersham Biosciences, Buckinghamshire, UK). Protein expression levels were analyzed with the ImageQuanT TL 1D gel analysis program (Amersham Biosciences).

### Statistical analysis

Data are presented as mean ± standard error (SE) of three or more separate experiments, as indicated. Normal data with equal variance were analyzed using Student’s t-test (for single comparison) or one-way analysis of variance (for multigroup comparisons) with Tukey’s procedure. Significance was assigned for *p* ≤ 0.05 as indicated.

## Additional Information

**How to cite this article**: Choi, H. *et al*. TGF-β Signaling Regulates Cementum Formation through Osterix Expression. *Sci. Rep*. **6**, 26046; doi: 10.1038/srep26046 (2016).

## Supplementary Material

Supplementary Information
