# Supplementary material for: TGF-β Signaling Regulates Cementum Formation through Osterix Expression
Source: Sci Rep. 2016 May 16;6:26046. doi: 10.1038/srep26046 (PMC4867644; doi:10.1038/srep26046)
Supplement: Supplementary Information [file srep26046-s1.pdf]

Supplementary Information

TGF-β Signaling Regulates Cementum Formation through Osterix Expression

Hwajung Choi[1], Yu-Hyun Ahn[1], Tak-Heun Kim[1], Cheol-Hyeon Bae[1], Jeong-Chae Lee[1], Hyung-Keun You[2], Eui-Sic Cho[1],*

[1]Cluster for Craniofacial Development and Regeneration Research, Institute of Oral Biosciences, Chonbuk National University School of Dentistry, Jeonju 54896, South Korea

[2]Department of Periodontology, School of Dentistry, Wonkwang University, Iksan 54538, South Korea

Supplementary Table 1. Primer sequences for ChIP-qPCR

Gene	Sense	Antisense
Alpl	GGCTGGGACAGACAGAATGT	CTGCAACAGGCAGGGTAAC
Osx	CTCATTGGATCCGGAGTCTTCT	TGTCTGTAGGGATCCACCCTCTA
Runx2	TGGTAGGCAGTCCCACTTTAC	GGCTGGTAGTGACCTGCAGAG

Supplementary Table 2. Primer sequences for real-time qPCR

Gene	Sense	Antisense
Tgfbr2	GAGAAGCCGCATGAAGTCTG	CATGAAGAAAGTCTCGCCCG
Osx	TCTCCATCTGCCTGACTCCT	AGCGTATGGCTTCTTTGTGC
Runx2	CCTCTGACTTCTGCCTCTGG	TAAAGGTGGCTGGGTAGTGC
Alpl	AGGGCAATGAGG TCACATCC	GCATCTCGTTATCCGAGTACCAG
Bsp	AAAGTGAAGGAAAGCGACGA	GTTCCTTCTGCACCTGCTTC
Ocn	ACCCTGGCTGCGCTCTGTCTCT	GATGCGTTTGTAGGCGGTCTTCA
Col1a1	CCGGAAGAATACGTATCACC	ACCAGGAGGACCAGGAAGTC
Col1a2	CAGCGAAGAACTCATACAGCC	CAGCGAAGAACTCATACAGCC
Dmp1	AGTGAGTCATCAGAAGAAAGTCAAGC	CTATACTGGCCTCTGTCGTAGCC
Gapdh	TGCCCAGAACATCATCCCT	GGTCCTCAGTGTAGCCCAAG

Legends for Supplementary Figures

Supplementary Figure 1. The original full-length blots of Figure 3C. The samples were derived from the same experiment and gels/blots were processed under the same experimental conditions. β-Actin was used as a loading control. Antibodies were purchased and used as follows. Anti-TβRII (Santa Cruz #sc-400, 1:500 dilution); Anti-Osx (Santa Cruz #sc-22536R, 1:500 dilution); Anti-Runx2 (Abcam #ab23981, 1:1000 dilution); Anti-β-Actin (Santa Cruz #sc-1616R, 1:2000 dilution). Protein markers were from SMOBIO (#PM2700) and/or BIO-RAD (#161-0374).

Supplementary Figure 2. Time-course of protein expression in OCCM-30 cells bearing the temporal sequence of cementoblast development in culture. (A) Western blot analysis of the indicated molecules in OCCM-30 cells treated with OM for the indicated durations. β-Actin was used as a loading control. The protein expression pattern shows distinct stages of cementoblast development, analogous to those of in vivo cementum formation as demonstrated by the distinct initial proliferation (Cyclin D1), intermediate differentiation (Osx, Runx2 and TβRII) and terminal matrix-formation (BSP and OPN) stages. (B) The original full-length blots of Supplementary Figure 2A were presented. The samples were derived from the same experiment and gels/blots were processed under the same experimental conditions. β-Actin was used as a loading control. Antibodies were purchased and used as follows. Anti-TβRII (Santa Cruz #sc-400, 1:500 dilution); Anti-Osx (Santa Cruz #sc-22536R, 1:500 dilution); Anti-CyclinD1 (Santa Cruz #sc-8396, 1:200 dilution); Anti-Runx2 (Abcam #ab23981, 1:1000 dilution); Anti-Opn (Abcam #ab8448, 1:1000 dilution); Anti-Bsp (Abcam #ab52128, 1:1000 dilution); Anti-β-Actin (Santa Cruz #sc-1616R, 1:2000

dilution). Protein markers were from SMOBIO (#PM2700) and/or BIO-RAD (#161-0374).

Supplementary Figure 3. OCCM-30 cells lacking functional TβRII (shTbr2) have altered biological properties. (A) Western blot analysis of TβRII in OCCM-30 cells stably expressing control shRNA (Con) and mouse *Tgfbr2* (Tbr2) after OM treatment for the indicated duration. β-Actin was used as a loading control. (B) Comparison of cell proliferation rates between control and shTbr2 OCCM-30 cells. The data represent the mean ± SE of three measurements of each group. **, $P<0.01$, *, $P<0.05$. (C) Representative SEM images of cultured control and shTbr2 OCCM-30 cells treated with GM or OM for 4 days. Scale bar, 50 μm. (D) The original full-length blots of Supplementary Figure 3A were presented. The samples were derived from the same experiment and gels/blots were processed under the same experimental conditions. β-Actin was used as a loading control. Antibodies were purchased and used as follows. Anti-TβRII (Santa Cruz #sc-400, 1:500 dilution); Anti-β-Actin (Santa Cruz #sc-1616R, 1:2000 dilution). Protein markers were from SMOBIO (#PM2700) and/or BIO-RAD (#161-0374).

Supplementary Figure 4. The original full-length blots of Figure 4A. The samples were derived from the same experiment and gels/blots were processed under the same experimental conditions. β-Actin was used as a loading control. Antibodies were purchased and used as follows. Anti-Osx (Santa Cruz #sc-22536R, 1:500 dilution); Anti-GFP (Abcam #ab290, 1:1000 dilution); Anti-β-Actin (Santa Cruz #sc-1616R, 1:2000 dilution). Protein markers were from SMOBIO (#PM2700) and/or BIO-RAD (#161-0374).

Supplementary Figure 5. The original full-length blots of Figure 5A. The samples were derived from the same experiment and gels/blots were processed under the same experimental conditions. β-Actin was used as a loading control. Antibodies were purchased and used as follows. Anti-Osx (Santa Cruz #sc-22536R, 1:500 dilution); Anti-Runx2 (Abcam #ab23981, 1:1000 dilution); Anti-DMP1 (TaKaRa #M176, 1:500 dilution); Anti-β-Actin (Santa Cruz #sc-1616R, 1:2000 dilution). Protein markers were from SMOBIO (#PM2700) and/or BIO-RAD (#161-0374). The two samples loaded right side have been excluded due to different experiment condition.

Supplementary Figure 1

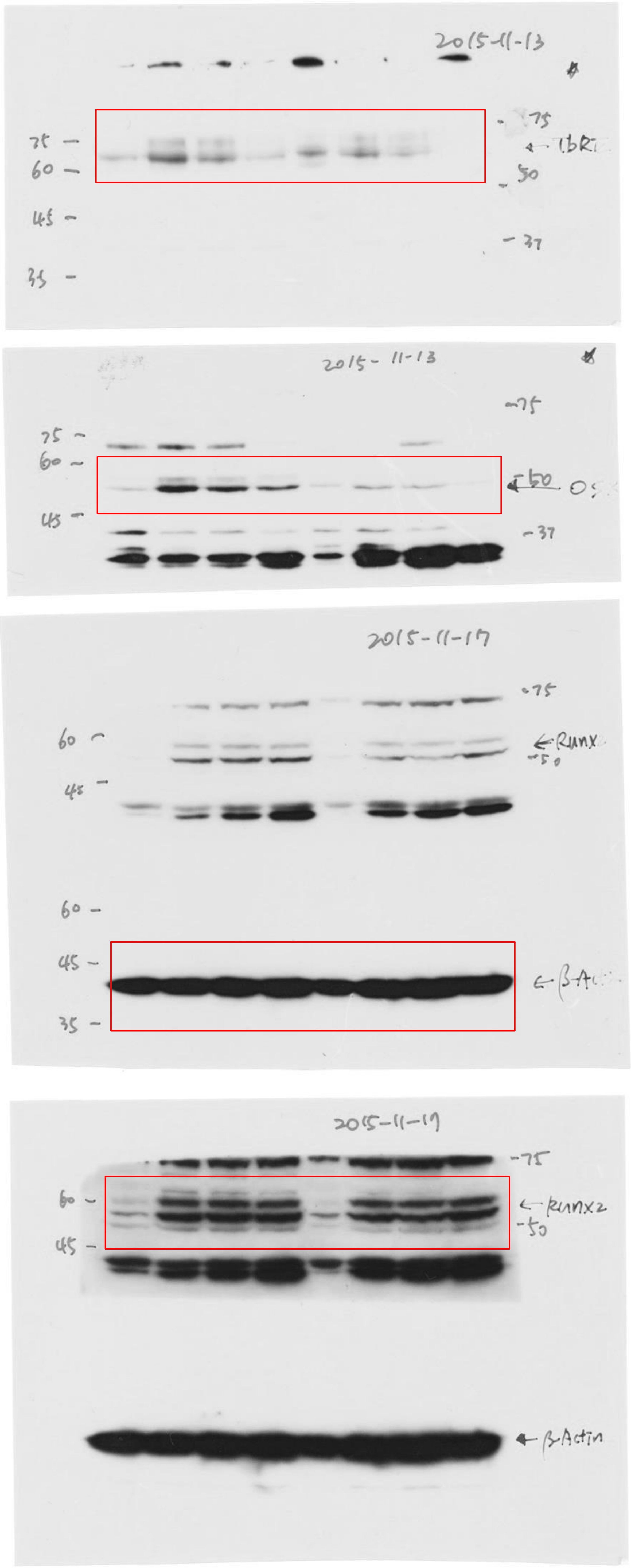

TβRII (65 kDa)

Osx (46 kDa)

Runx2 (short exposure) (57 kDa)

β-Actin (42 kDa)

Runx2 (57 kDa)

β-Actin (long exposure) (42 kDa)

Supplementary Figure 2

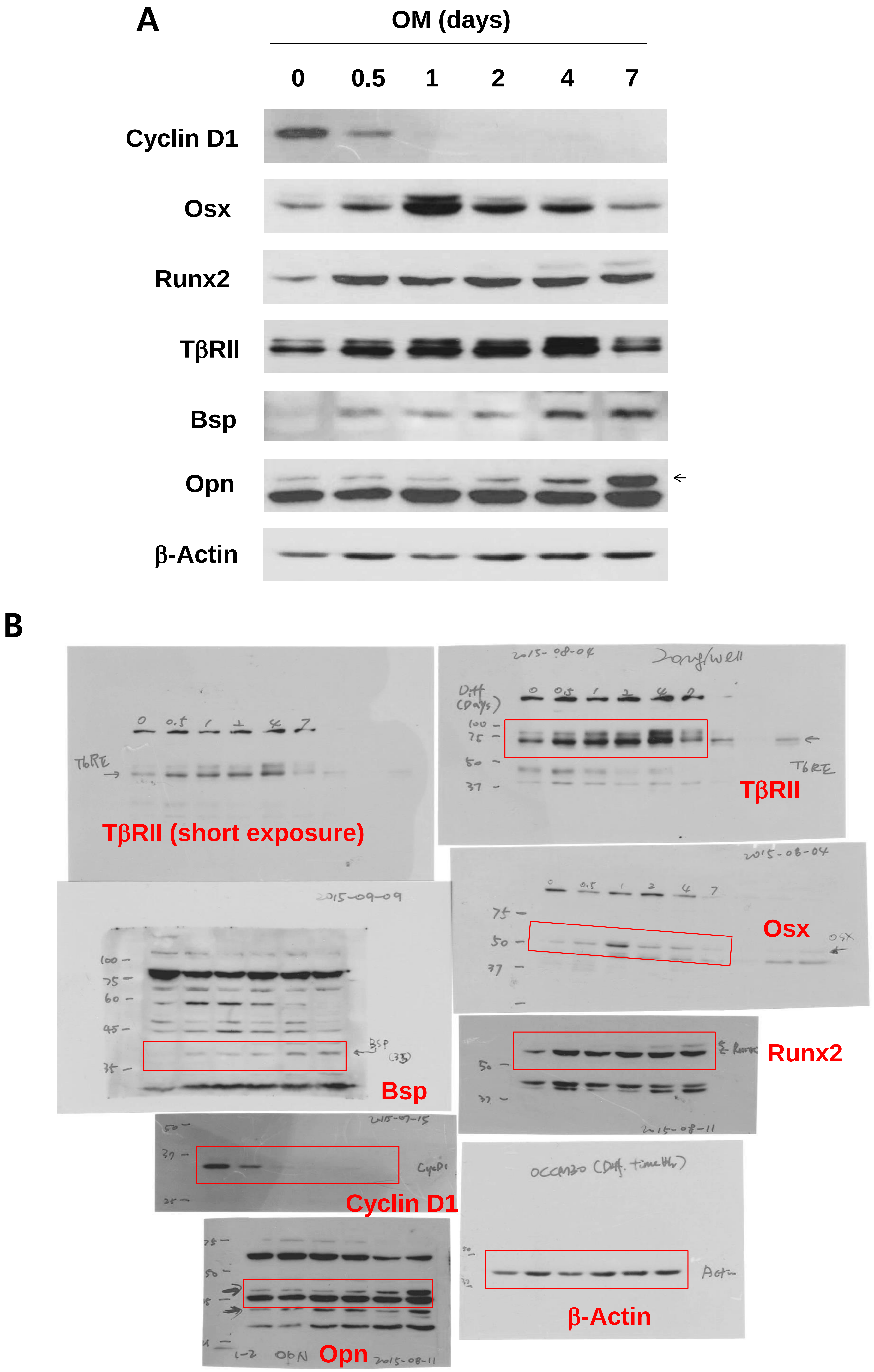

Supplementary Figure 3

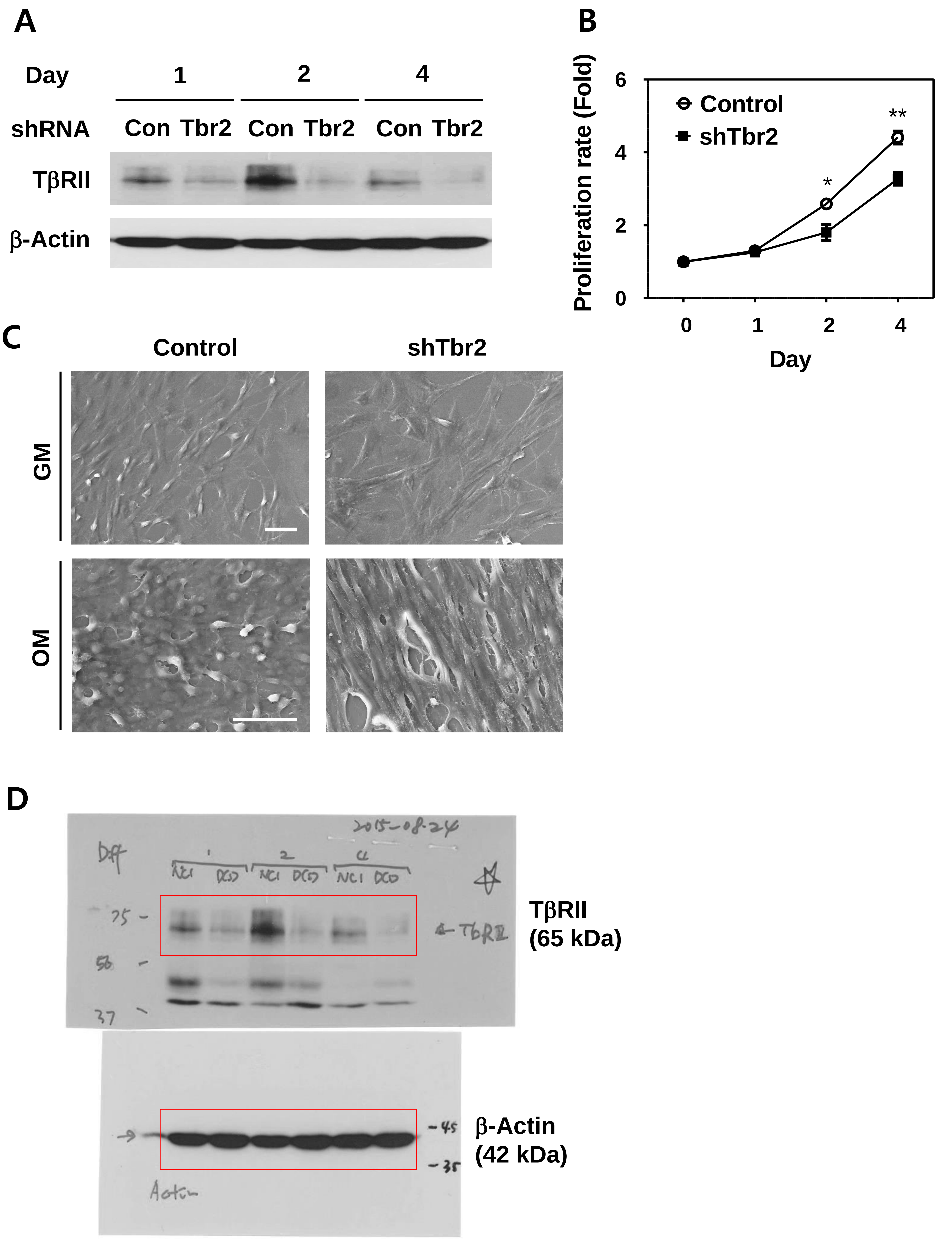

Supplementary Figure 4

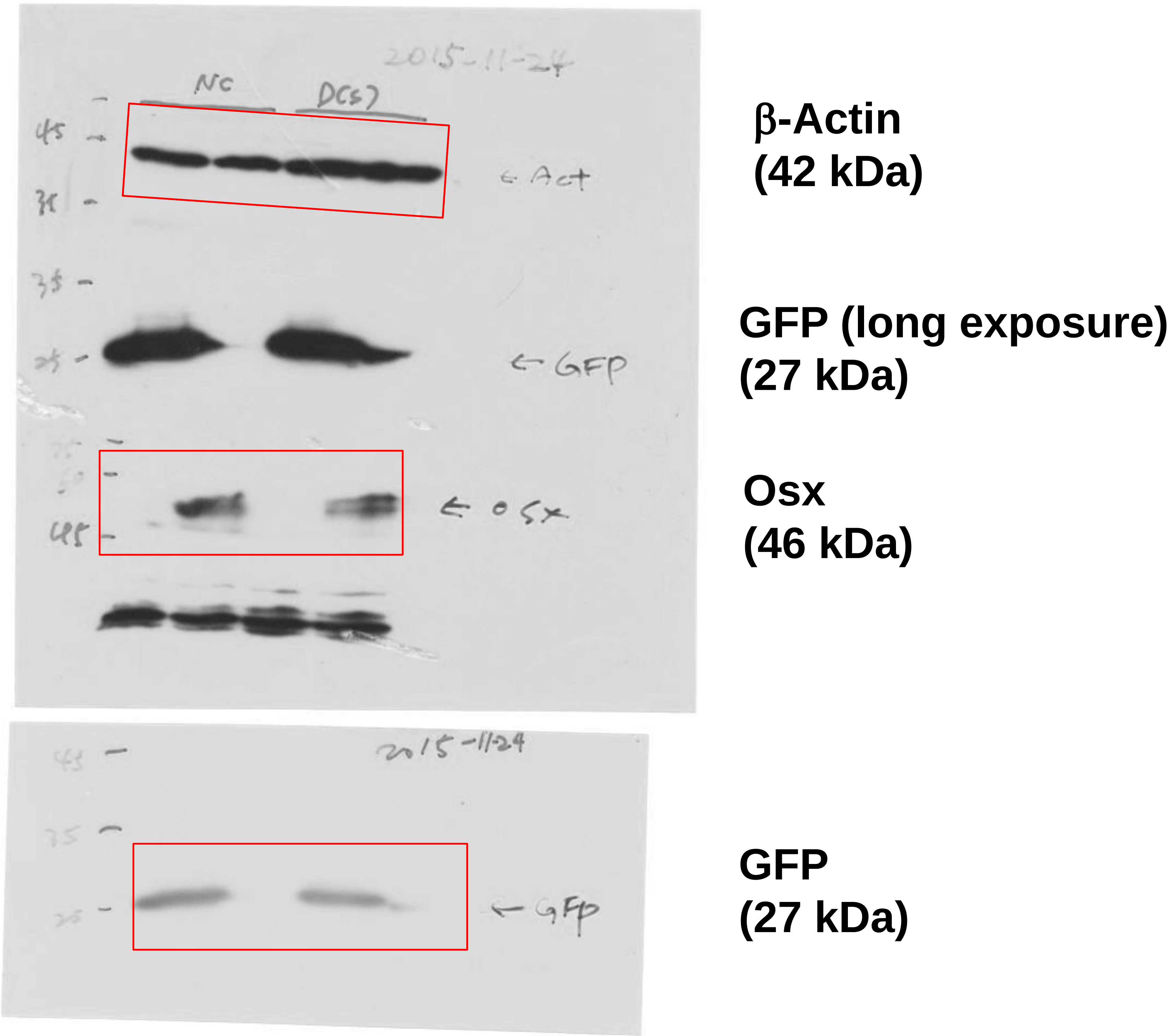

Supplementary Figure 5

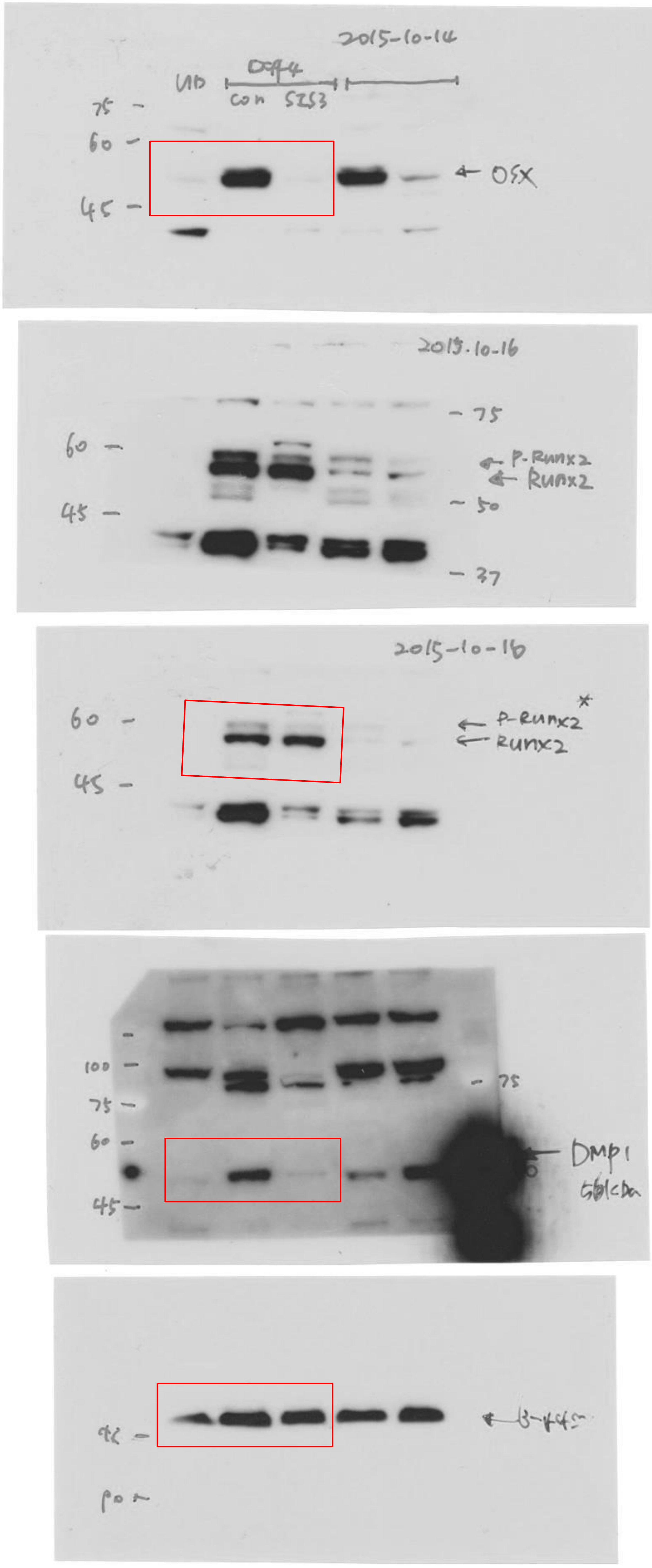

Osx
(46 kDa)

Runx2 (long exposure)
(57 kDa)

Runx2
(57 kDa)

DMP1
(56 kDa)

β-Actin
(42 kDa)
